# Factors That Influence Successful Adoption of Real-Time Location Systems for Use in a Dementia Care Setting: Mixed Methods Study

**DOI:** 10.2196/45978

**Published:** 2024-04-08

**Authors:** Lynn Haslam-Larmer, Alisa Grigorovich, Leia Shum, Andria Bianchi, Kristine Newman, Andrea Iaboni, Josephine McMurray

**Affiliations:** 1 KITE Research Institute Toronto Rehabilitation Institute University Health Network Ontario, ON Canada; 2 Recreation and Leisure Studies Brock University St. Catherines, ON Canada; 3 Dalla Lana School of Public Health University of Toronto Toronto, ON Canada; 4 Centre for Clinical Ethics Unity Health Toronto Toronto, ON Canada; 5 Department of Psychiatry University of Toronto Toronto, ON Canada; 6 Lazaridis School of Business & Economics Wilfrid Laurier University Brantford, ON Canada

**Keywords:** remote sensing technologies, dementia, real-time location systems, Fit between Individuals, Tasks, and Technology framework, FITT framework, technology implementation

## Abstract

**Background:**

Technology has been identified as a potential solution to alleviate resource gaps and augment care delivery in dementia care settings such as hospitals, long-term care, and retirement homes. There has been an increasing interest in using real-time location systems (RTLS) across health care settings for older adults with dementia, specifically related to the ability to track a person’s movement and location.

**Objective:**

In this study, we aimed to explore the factors that influence the adoption or nonadoption of an RTLS during its implementation in a specialized inpatient dementia unit in a tertiary care rehabilitation hospital.

**Methods:**

The study included data from a brief quantitative survey and interviews from a convenience sample of frontline participants. Our deductive analysis of the interview used the 3 categories of the Fit Between Individuals, Task, and Technology framework as follows: individual and task, individual and technology, and task and technology. The purpose of using this framework was to assess the quality of the fit between technology attributes and an individual’s self-reported intentions to adopt RTLS technology.

**Results:**

A total of 20 health care providers (HCPs) completed the survey, of which 16 (80%) participated in interviews. Coding and subsequent analysis identified 2 conceptual subthemes in the individual-task fit category, including the identification of the task and the perception that participants were missing *at-risk* patient events. The task-technology fit category consisted of 3 subthemes, including reorganization of the task, personal control in relation to the task, and efficiency or resource allocation. A total of 4 subthemes were identified in the individual-technology fit category, including privacy and personal agency, trust in the technology, user interfaces, and perceptions of increased safety.

**Conclusions:**

By the end of the study, most of the unit’s HCPs were using the tablet app based on their perception of its usefulness, its alignment with their comfort level with technology, and its ability to help them perform job responsibilities. HCPs perceived that they were able to reduce patient search time dramatically, yet any improvements in care were noted to be implied, as this was not measured. There was limited anecdotal evidence of reduced patient risk or adverse events, but greater reported peace of mind for HCPs overseeing patients’ activity levels.

## Introduction

### Background

The increasing demands for efficiency and improvements in the delivery of care have prompted interest in the use of tracking technologies as a solution to monitor the movements of patients, providers, and equipment across health care settings, including hospitals and long-term care homes. As an example of this type of technology, real-time location systems (RTLSs) can be used to identify the physical location of people or assets across time and space [1,2]. RTLS technologies are local positioning systems that typically consists of a wireless device attached to objects or worn by people, with environmentally embedded reference points that receive wireless signals from these wireless devices. Software connected to this wireless network can provide continuous real-time visualizations of the location data on a facility map. RTLS installations have been studied in a wide variety of health care settings to monitor individuals’ movements (eg, residents or patients and health care providers [HCPs]) [3-5] and assets (eg, surgical equipment) [6] and, more recently, to collect health data [3,7-9]. These systems are particularly well suited for monitoring movement and reducing the risk of unaccompanied exit (eg, elopement); however, RTLS data have also been used to inform clinical decision-making processes and to monitor health status or the effects of an intervention [10]. Similar systems using wireless geolocation have already been successfully implemented in other sectors, such as insurance [11] and telecommunications [12], but present novel challenges when deployed in health care settings, particularly when they are used to generate personal and life-space data from vulnerable populations [13,14].

In the context of dementia care, interest in RTLS has been driven by the desire to evaluate cognition and health status more objectively and to enable the prediction of patient decline from changes in patterns of movement [7]. Other potential benefits of RTLS may include allowing greater freedom of movement within secure units and reducing the risk of unaccompanied exits (eg, elopement). A recent review by Grigorovich et al [2] identified barriers and enablers to implementing RTLS in people with dementia. Barriers to implementation included the following: a lack of motivation for providers to engage due to issues, such as perceptions of low value in use; technology infrastructure and maintenance challenges, such as a lack of on-site technical support and maintenance; and myths and stories about the technology and its purpose that are shared informally due to a lack of understanding about the systems and poor communication about how they work. In contrast, enablers of implementation included the following: being sensitive and adapting to local workflows, policies, and technologies; usability and user-centered design of the RTLS system; frequent communication with care providers; and establishing policies, frameworks, governance, and evaluations to assess the utility and improve the quality of the installation. The review found a striking lack of evidence to support the use of RTLS technologies to improve the quality of residents’ lives or the workflow of HCPs. RTLS adoption in acute care settings has been slow, with concerns regarding provider privacy pitted against the goal of patient safety and efficiency [3].

### Objectives

While many technology adoption models are in use [4] and have provided considerable insight into the adoption of digital technologies in the health care domain [5,6], to date, no study has specifically examined the implementation of an RTLS in a tertiary dementia care setting. To better demonstrate the potential barriers and enablers of RTLS adoption in a clinical setting, we used the Fit Between Individuals, Tasks, and Technology (FITT) framework developed by Ammenwerth et al [8]. The FITT framework suggests that the adoption of new technology in a clinical environment will depend on the *fit* between the attributes of the users (eg, computer literacy and education), the attributes of the technology (eg, user interface and functionality), and the attributes of the clinical task (eg, degree of complexity and workflows) [8]. The framework can be used predictively [9] or retrospectively to identify “deltas” between the expectations of a technology’s implementation in a clinical setting and its actual relevance in the workflows and to its users [15]. The FITT framework’s strength is its emphasis on the interaction between the individual and the task, with the understanding that harmonization will positively impact the implementation and adoption of novel technologies. In this study, we aimed to identify the factors that influence the adoption or nonadoption of an RTLS during its implementation in a secure inpatient unit for persons living with dementia in a rehabilitation hospital.

## Methods

### Study Design

This partially mixed methods concurrent study [16] included a brief preinterview survey followed by an in-depth semistructured interview. We chose this study design due to the exploratory nature of the research and the combination of quantitative and qualitative data that provides a deeper and more comprehensive understanding of the topic [17,18].

### Study Setting

This study was conducted in a 20-bed secure inpatient dementia care unit of a large urban rehabilitation hospital in Ontario, Canada. Patients were admitted primarily from long-term care homes to receive specialized assessment and a personalized care plan to manage agitation, aggression, and other behaviors that interfere with the quality of life and safety of individuals living with dementia and their caregivers (eg, formal and informal). The unit comprised a team of interprofessional care providers (eg, nurses, physiotherapists, occupational therapists, recreation therapists, and geriatric psychiatrists) with expertise in addressing the range of physical, mental, and emotional challenges often associated with dementia. As the unit was a secure unit, the team was familiar with using technology to help monitor patients’ health status and movements. For instance, the unit used the WanderGuard elopement prevention system (Securitas Healthcare), which alerted providers if patients attempted to leave the unit unaccompanied.

### Participants

Participants in this study were HCPs who regularly worked on the unit where the technology was implemented (eg, nursing aides, nurses, allied health professionals, and unit leadership). There were approximately 40 frontline staff members who interacted with patients, in addition to an advanced practice nurse and a unit manager. HCPs working on the unit were exposed to the RTLS and were individually able to decide to engage with the technology. The participants were recruited via an email distributed by the unit manager to all HCPs and by word of mouth during training sessions, team meetings, and daily report huddles.

### RTLS Intervention

The implemented RTLS was a commercially packaged ultrawideband live monitoring system installed to locate and track patients on the unit. The hardware consisted of wall-mounted beacons that created a local Wi-Fi mesh and wearable tags that provided location data within the networked area. The wearable tags were fitted to patients on the unit as nonremovable bracelets. All participants’ substitute decision makers were provided with an opportunity to consent (or refuse) to have the patient wear the bracelet and to separately consent (or refuse) to have the location data collected and stored for research purposes (eg, development of clinical algorithms).

In the nursing station, a tablet app provided a view-only dashboard of the unit map and a live feed of patient locations. The tablet was locked to “kiosk mode” after log-in, thus preventing the app display from timing out. We held in-service training sessions to familiarize HCPs with the app’s layout and functions, including how to read the map and search for patients. Although the RTLS can be used in various ways (eg, nursing call bell), no other features were enabled in this study; the system was exclusively used to locate patients. The RTLS location data were stored on a secured and sectioned client cloud server approved by the Health Insurance Portability and Accountability Act.

### Ethical Considerations

This study was approved by the University Health Network (UHN) Research Ethics Board (#20-6277) and the Wilfrid Laurier University Research Ethics Board (ID# 6767) and was conducted in accordance with the principles of the Declaration of Helsinki. The survey participants reviewed a web-based consent form, had an opportunity to contact the study team with any questions, and indicated their informed consent to participate through the UHN REDCap (Research Electronic Data Capture; Vanderbilt University) e-consenting process. Then, a personalized link was sent from the REDCap system via email for participants to complete the preinterview survey. Upon completion of the interview, HCPs received a gift card valued at CAD $20 (US $14.8).

### Data Collection

Following installation, a research study team member (LS) held training sessions for HCPs to explain how the bracelets worked to track patient location and how to use the nursing station tablet and app. During the training sessions, HCPs were verbally informed that they would receive an email inviting them to participate in the study. Approximately 6 weeks after installation, HCPs were invited via email to participate in the study, which included a short survey followed by a more in-depth, semistructured interview. The survey (facilitated via the UHN-managed REDCap servers) took approximately 5 minutes to complete and included questions regarding their sociodemographic characteristics and attitudes toward the use of technology when caring for patients with dementia. These attitudinal questions asked HCPs regarding their perceptions of the following: (1) their comfort with technology generally and location-tracking technology in particular, (2) the appropriateness of using location-tracking technology on patients with dementia, (3) whether they planned to use the location-tracking system installed in their unit, and (4) the appropriateness of technology to monitor HCPs. Furthermore, staff were invited to provide free-text comments regarding why they planned to use or not use the technology. After completing the survey, the participants were contacted to participate in the semistructured phone interview. The interview comprised 12 primary questions (Multimedia Appendix 1), asking about their past experience (if any) using RTLS, expectations and goals of the system, user experiences with the system, and how they perceived the system to impact their care of patients. The interviews lasted approximately 1 hour and were audio recorded. The interviews continued until saturation was achieved.

### Data Analysis

Qualitative analysis was conducted using NVivo (version 12; Lumivero). Anonymized interviews were transcribed verbatim. Transcripts were read by all the research team members (LHL, AG, JM, KN, and AB) to gain a comprehensive understanding of the content. Several team meetings were held to review the transcripts and develop a coding strategy. We used inductive and deductive analytic techniques, such as systematic coding and constant comparison to fit data with existing literature and to identify conceptual categories and insights [19]. After the first round of independent coding, the team met to ensure the optimal categorization of utterances within themes and subthemes. Each interview was coded by at least 2 team members. Group discussions and consensus were used to resolve any disagreements in coding.

Our deductive analysis of the interview data was informed by the FITT framework developed by Ammenwerth et al [8] and included the following categories: (1) individual and task, (2) individual and technology, and (3) task and technology. The FITT framework suggests that the adoption of new technology in a clinical environment will depend on the “fit” between the attributes of the individuals (eg, computer literacy), the attributes of the technology (eg, user interface and functionality), and clinical task attributes (eg, degree of complexity) [8]. The individual category represents not only the individuals using the technology but also groups of users (eg, HCPs) and considers constructs such as the physical settings in which they operate, thus representing any nontask phenomenon that may influence uptake and use of the technology. The technology category includes components of a digital application, such as hardware, software, and network infrastructure, or analog tools, such as paper-based care plans or manuals, used to complement a particular technology. The task category represents the clinical work and work-related processes that occur within a particular care setting.

## Results

### Participant Demographics

A total of 20 HCPs completed the preinterview survey. The survey’s primary purpose was to characterize the participant sample and allow the study team to determine whether the diverse range of HCPs on the unit was represented in the interview process. Of the 20 HCPs who completed the survey, 16 (80%) completed interviews, a sample size deemed sufficient given the exploratory nature of the single-site study and relative professional homogeneity of the sample population that allowed us to achieve saturation (Table 1) [20]. The participants who completed the survey and interview were predominantly female individuals (16/20, 80%), aged <50 years (16/20, 80%), and provided direct patient care (eg, nursing and support staff and allied health staff; 14/20, 70%).

**Table 1:** Participant demographics (n=20).

Measure and item			Surveys (n=20), n (%)		Interviews (n=16), n (%)
**Sex**					
	Female	16 (80)		14 (88)	
	Male	4 (20)		2 (12)	
**Age group (y)**					
	≥25 to <35	6 (30)		5 (31)	
	≥35 to <50	10 (50)		8 (50)	
	≥50	2 (10)		2 (12)	
	Did not answer	2 (10)		1 (6)	
**Education**					
	College, other nonuniversity certificate, or diploma	7 (35)		5 (31)	
	Bachelor’s degree	10 (50)		8 (50)	
	Master’s degree	3 (15)		3 (19)	
**Occupation**					
	Providing direct patient care (nursing and support staff)	14 (70)		10 (62)	
	Allied health (eg, OT^a^ or PTA^b^ and recreation therapy)	4 (20)		4 (25)	
	Leadership or administration	2 (10)		2 (12)	
**Work experience** **(y)**					
	≤1	3 (15)		3 (19)	
	2 to 5	1 (5)		0 (0)	
	6 to 10	3 (15)		2 (12)	
	>10	11 (55)		10 (62)	
	Did not answer	2 (10)		1 (6)	
**Current** **position** **(y)**					
	≤1	7 (35)		7 (44)	
	2 to 5	4 (20)		3 (19)	
	6 to 10	3 (15)		1 (6)	
	>10	4 (20)		4 (25)	
	Did not answer	2 (10)		1 (6)	

^a^OT: occupational therapist.

^b^PTA: physiotherapy assistant.

All the participants who completed the survey agreed or somewhat agreed with the statements that they were “satisfied with their job,” were “confident in their ability to learn a new technology,” and were “familiar with location-tracking technology and how it works” and “agreed” or “somewhat agreed” that location-tracking technology was “acceptable when tracking the movements of persons living with dementia” (Table 2). There was less concordance of opinion regarding the use of location-tracking technology to monitor the movement of providers; 12 (60%) of the 20 respondents reported that its use would be “somewhat or completely unacceptable,” and 4 (20%) reported its use as “somewhat or completely acceptable” (Tables 3 and 4).

**Table 2:** Survey question responses (N=20).

Questions	Agree, n (%)	Somewhat agree, n (%)	Neither disagree nor agree, n (%)	Somewhat disagree, n (%)	Disagree, n (%)
“I am satisfied with my job.”	15 (75)	5 (25)	0 (0)	0 (0)	0 (0)
“Overall, I find technology is useful in my job.”^a^	15 (79)	1 (5)	3 (16)	0 (0)	0 (0)
“I feel confident in my ability to learn how to use new technology.”	17 (85)	3 (15)	0 (0)	0 (0)	0 (0)
“I am familiar with location-monitoring technology and how it works.”	14 (70)	6 (30)	0 (0)	0 (0)	0 (0)

^a^n=19.

**Table 3:** Survey question responses (N=20).

Questions	Completely acceptable, n (%)	Somewhat acceptable, n (%)	Neither acceptable nor unacceptable, n (%)	Somewhat unacceptable, n (%)	Completely unacceptable, n (%)
“In general, I believe that using location-monitoring technology to track the movements of persons with dementia is”	16 (80)	4 (20)	0 (0)	0 (0)	0 (0)
“In general, I believe that using location-monitoring technology to track the movements of health care provider is”	1 (5)	3 (15)	4 (20)	7 (35)	5 (25)

**Table 4:** Survey question responses (N=20) to “I plan to use the location-monitoring technology (referred to as “RTLS^a^”) during the pilot study at the SDU^b^.”

Responses	Value, n (%)
Always	10 (50)
Often	5 (25)
Sometimes	4 (20)
Rarely	1 (5)
Never	0 (0)

^a^RTLS: real-time location system.

^b^SDU: special dementia unit.

### Interview Results

Interview data were analyzed and reported in 3 categories that correspond to the FITT framework [8]. The quality of the fit between these constructs depends on each of their characteristics and their alignment and the ability of management or the technology adoption team to influence the adaptation of the task, the technology, or the individual to improve the quality and success of the implementation.

### Individual-Task Fit

#### Overview

This category represents individual attributes, including individual users, user groups, the organization, and the working processes involved in completing the task, which in this case was using the RTLS to locate a patient in the unit. The ostensibly simple task of locating patients within a locked unit is complicated, given that many are mobile and some exhibit motor agitation, where restlessness keeps them in constant motion [21]. Using coding and subsequent analysis, we identified 2 conceptual subthemes: (1) characterization of the task and (2) the association between locating and monitoring patients for HCPs.

#### Characterization of the Task

In the first subtheme, participants identified the characteristics of the task, particularly the time-sensitive nature of some activities; for example, medication administration, which benefits from real-time monitoring, or conversely, the inability to locate a patient promptly when needed. One provider noted that the task of locating patients was more complicated with this specialized population as follows:

Because our patients are confused, and they have no concept of their own place or their own room, majority of them, they wander around the unit.

P5

Legacy closed-circuit television cameras that streamed video in real time to monitors located at the nursing desk only allowed the visualization of public spaces (eg, hallways). A provider articulated the difficulty with locating a patient using this existing system, as follows:

[T]here’s no cameras in the patient rooms. Only in the hallway. Like [in] the public spaces...but not in the patient room. So, if you can’t see them in like the more public spaces, then you would actually have to get up and search every single room because the patients might not be in their room...They could be in someone else’s room, they could have fallen asleep on someone else’s bed, maybe they’re in someone else’s bathroom.

P21

Furthermore, many HCPs shared that the task of locating patients manually made other tasks, such as medication administration, more complex:

Like if you wanted to give medication, [if] we couldn’t find, um, patients, we have to go and look for them in room by room.

P6

Moreover, we conducted this study during the COVID-19 pandemic, during which patients who tested positive or were symptomatic of infection had to be isolated on the unit. While external doors were locked, most internal doors were not, particularly in patient rooms. The task of maintaining isolation for new patients, those who were exposed, or those who tested positive for COVID-19 in this unit was challenging, as they had difficulty remembering and understanding the need for isolation and physical distancing. HCPs had to monitor patients every 15 minutes to ensure that they stayed isolated and had to use a variety of strategies to encourage patients to remain in their rooms (eg, placing stop-strip door banners across the unit and stop signs on closed doors and sitting outside their rooms). Hallway cameras were perceived as inadequate for the task of supporting isolation, and providers felt that they did not allow for an anticipatory response when a patient was either moving from their bed or toward a restricted hallway or room.

In addition, this theme included the individuals’ need to track items other than human or physical assets in a dynamic space. One HCP stated that putting tags on nurses could help them locate each other when one of their patients required assistance. Another suggested that they would be useful for quantifying direct care hours provided by different providers.

Similarly, some participants suggested that the RTLS may be helpful for monitoring assets such as patient’s phones and walkers that are often misplaced. This is particularly important for assistive technologies, such as walkers, that support mobility and activities of daily living. One HCP noted as follows:

Someone who always uses their walker or, you know, always misplaces it...especially a lot of our patients now have, you know, like, personal items such as cell phones...and they’ve been misplaced and we’re always looking for it.

P8

Especially with our...patient...group right now, a lot of them have their own cell phones. A lot of them, they put it in their pockets and a lot of the provider don’t really track where they are. So, when the patients are looking for it, we’re the ones who have to go around and look, you know, in the bag, in the laundry, in their closet.

P8

#### Association Between Locating and Monitoring Patients for HCPs

The second individual-task subtheme accounted for an HCP’s perception that the act of locating or knowing the location of a patient on the unit was strongly associated with a better standard of care by helping to mitigate potentially “risky” events. One HCP noted as follows:

It gives me more stress if every time I keep on looking for my patient, I couldn’t get my things done right away, ‘cause I have to find them and always making sure that they’re safe.

P12

Being unable to identify when one patient entered another patient’s room was a common concern noted by HCPs:

So once in a while, we do our rounds, but if a patient sneaked into somebody’s room, how would I know, until I really look at who is in bed?

P5

We cannot find them because they wander around, you know, they...pace and...they are so intrusive, they go to other, uh, patients’ rooms.

P6

A number of HCPs noted that “distractions” would often interrupt the process of physically locating a patient on the floor, for example:

Another patient come(s) up to you and need(s) something, then you have to find someone else to help you.

P21

I would be finding one patient and then I would be distracted and go to another nurse who needed me.

P14

The same HCP mentioned that the task is never just a “straight search” and that their presence on the floor would be noted by other patients and staff, and they would be drawn into monitoring or supporting interactions in support of patients or their colleagues.

### Task-Technology Fit

#### Overview

This category of the FITT framework represents the working processes involved in completing the task (locating a patient) and how they interact with the RTLS (consisting of the bracelet, the wall-mounted beacons receiving the ultrawideband signal, the app on the tablet at the nursing desk, and the software application). The task-technology fit category consisted of three subthemes: (1) reorganization of the task, (2) personal control in relation to the task, and (3) efficiency and resource allocation.

#### Reorganization of the Task

Reorganization of the task was expressed by HCPs as the ability to locate and monitor patients in real time without having to go into each patient room. HCPs using the technology started their search at the nursing station, where they interacted with the tablet to identify the location of the patient and then proceeded directly to the patient, rather than conducting a random search. Providers found this beneficial for several reasons, including using the tablet to “spot-check” to see where a patient was or being able to directly find a patient when they wanted to:

The purpose as I see it, is to be able to locate the patient when you need to locate a patient, whether it’s because of double checking that they haven’t eloped or you’re trying to locate them for a therapeutic reason or for you know, I think this is the big one was like family members coming to visit and they wanted, you know, to find their loved one.

P4

Furthermore, we found evidence of evolving work routines or stable actions that achieved work across time and space through adaptive routinization that supported HCPs use and adoption of the technology. However, adjustment of the technology (eg, tablet number and location) or routine (eg, shift change and anticipating interpatient altercations) to achieve a successful implementation was still clearly a work in progress at the time the interviews were conducted:

It’s been so new, it’s hard for us to remember that we have this...the more people are talking about it, the more that we’re remembering, “Oh we could use this instead of you know, running about the unit searching for patients.”

P3

Habitually, I would start the lap- and then realize that I’m wasting time, cut through the care station, find them on the monitor and then, yeah. So, it-it-it did change the fact that I-I wouldn’t do more than one lap before realizing that I could go use it.

P8

Right at shift change, I’m not gonna go in there and push through the nurses, trying to do their shift change to look at the monitor. I’ll just do a couple extra laps until I find them because it’s a reasonably small unit.

P8

#### Personal Control in Relation to the Task

The second subtheme in the task-technology fit category was the recognition of personal control in relation to the task of locating patients. The HCPs perceived that they had more control by being able to decide how and when to find patients using the app. For example, if they were in the nursing station performing another task, they were also able to see or locate a patient at a glance on the tablet. Examples of this narrative are as follows:

Instead of walking around three times around the entire unit, I know exactly where my patient is. I also can monitor them, uh, remotely.

P5

Especially, uh, at night-time it’s a lot better because, uh, we know that, uh, which patient is, uh, in the room and which one is out. When we are sitting in the nursing station, we can say like this...patient has come out of the room and is in a hallway or he has woken up.

P6

At night, like I wanna see which patients are asleep, or awaken, are awake, like, if the bed alarms don’t work.

P13

#### Efficiency and Resource Allocation

Efficiency and resource allocation were identified as a subtheme in the task-technology fit category. The HCPs stated that they were saving time and resources through their ability to find a patient directly using the app. They shared that a room-by-room search of the unit could potentially take up to 15 minutes each time as their patients were very mobile, which was challenging as they were also likely to be distracted by another patient during their search:

It saves me time from going from room to room because these patients, normally they walk around and go to other people’s rooms so where you least expect them to be that’s where they sometimes are. And, um, if you think they’re gonna be in their room, most times you’re wrong. They’re in somebody else’s room away from-- Maybe the other corner, you know, so it saves your time when you use this device. You located them faster.

P11

HCPs further reflected that the time they saved using the app to locate patients could potentially be used for accomplishing other tasks. For example, 2 providers stated as follows:

When time is saved, then I can invest that time more on patient care, and all the things that I can do with extra time.

P13

You still have to physically go look for the patient, but at least it does narrow it down for you.

P4

However, the location of a singular tablet installed at the nursing station may pinpoint a patient in 1 location only to find that they had moved by the time the HCPs reached there. Finally, the technology was considered helpful in locating multiple patients when the providers needed to run a group activity:

If I run groups, and I want to get, you know, specific patients of a certain group—um, then I would, you know, go to iPad and see where they are.

P8

### Individual-Technology Fit

#### Overview

Individual-technology fit represents the relationship between the attributes of the individuals and the attributes of the technology and, in this study, comprised the most populous coding category for the interview data. The “individual” construct in the FITT model represents the HCP, who uses the technology to locate a patient; the patient, who wears the bracelet; the users as a group; and the organization, in which the technology is installed and the “tasks” and work processes are occurring. A total of four subthemes were identified in the individual-technology fit category: (1) privacy and personal agency, (2) trust in the technology, (3) user interfaces, and (4) perceptions of increased safety.

#### Privacy and Personal Agency

The subtheme of privacy and agency has multiple perspectives, given that while HCPs were the “users” of the technology, patients were the ones wearing the bracelet and being tracked. In alignment with the survey data, HCPs felt that patients on the unit had a limited right to privacy due to their perspective that these patients required close supervision to reduce their risk of harm (eg, preventing them from a fall or an altercation with another resident). This perception was expressed in narratives that suggested that patients with dementia lose their right to privacy (and conversely, their right to refuse to be monitored using the bracelet) as their cognitive impairment and behavior presents a risk to themselves, their family, and the organization:

There is no privacy for patients with dementia, with behaviors...privacy means that you are putting them in, uh, at risk.

P6

However, HCPs recognized that other patients (eg, those without dementia) had a right to privacy and may thus find the use of RTLS to monitor them unacceptable:

I don’t know about the patients on other floors if...it’s monitoring every move...they don’t wanna be like, to know that they’ve gone to the washroom, you know? These just—these are- these are things they might not like.

P6

Despite HCPs belief that patients in the unit waived their right to privacy in return for what they believed to be better care and safety, in general, providers believed they were “entitled to their own privacy” (P16) and did not agree with the concept of using location tracking for HCPs. In particular, HCPs were worried that if the ability to track staff was initiated by management, it could be used to sanction them:

So, tracking staff, they would be depending on the culture, and I think just general trust of senior leadership. Like, are you tracking if I’m taking too many breaks, if I’m doing my job correctly?

P21

Those who personally felt that there may be value in tracking the movements of staff acknowledged that other HCPs might not agree but variously supported the tracking of providers to help locate each other, gather supporting data as it relates to workload monitoring, and build more efficient units. For example, they suggested that tracking providers’ movements may be useful for showing how much they move in a day, how often they are in patient rooms, how many staff members are required to assist a patient, and for how long:

Cause I think we don’t estimate that correctly.

P22

The potential risks associated with the collection and storage of patients’ data were not a focus of concern; providers did not reflect on data use and storage until prompted during the interviews. Furthermore, providers made assumptions that the location data were kept on internal hospital servers; while for this study all data were stored on hospital secure servers, this is unusual and had to be negotiated with the equipment vendor who retained data on proprietary servers. One HCP stated as follows:

I would hope the security, or the data is secure and, um, only, you know, used for the study purposes.

P3

Another wondered if the data could be used for alternative purposes that they were not aware of:

I don’t know that information is being used for some other sinister reason I don’t know. Like whether they trust us with that information.

P4

#### Trust in the Technology

The subtheme of an individual’s trust in the technology relates to the reliability of the system—that the technology was working when required—and how it was used or adopted as a result. However, HCPs displayed a tolerance for unreliability in this product, likely because there were workarounds; that is, the providers could revert to walking the unit to find a patient, which made the consequences of unreliability minor:

Okay. So, you have a little bit of a clue that there’s something wrong with the bracelet if it’s not moving then?

Interviewer

Yeah. Especially when the bracelet, uh, is on the screen in one location and patient is physically on a, in a completely different location.

P5

Okay. Do you think that it not working, um, will influence how you trust the system or how you use it?

Interviewer

Uh, no, because it doesn’t happen often, it’s just once in a while malfunction.

P5

#### User Interfaces

The individual-technology fit category includes a subtheme of how patients and HCPs interfaced with the technology. Many HCPs shared that some patients “fidgeted” with or attempted to remove the bracelets. When asked to expand on this, HCPs explained that “some patients do remove, like they have catheter or other bracelets, they remove it. It’s just, uh, one of their behaviors” (P13). In response to a patient removing their RTLS bracelet or trying to remove it, providers reported that they would attempt to redirect the patient or move the bracelet to another location on their body (eg, an ankle) despite acknowledging that such attempts to remove the bracelet might be an expression of their desire to not wear the bracelet at all:

Sometimes we don’t understand, but of course they’re showing that they don’t like it.

P15

Despite acknowledging this, HCPs did not explore the reasons why patients attempted to remove their bracelet. However, one provider did report that they would remove the bracelet if “the patient states exactly that he knows what the bracelet is and he states like, ‘I don't want to be monitored’” (P13).

Furthermore, providers mentioned that the patient-technology interface was a tool for maintaining patients’ dignity while wearing the bracelet, considering their lack of agency over whether they wore the tracking device. The bracelet esthetic mimicked something other than a surveillance device. For example, one provider reported that a patient “referred to the bracelet...as their Apple watch and it was kind of cool that they had a nice new watch, which was beautiful” (P07). She suggested that this was beneficial and stated as follows:

In the future, if it could even mimic a watch or have a watch face on it, that it, you know, when it goes into screensaver or whatever becomes a watch face, then it would be even more patient-friendly, user-friendly, right?”

P7

Similarly, another HCP stated as follows:

Even people with dementia want to feel ready for the day and wear important pieces like a watch, carry a wallet. Wearing such pieces give dignity and acknowledges that people still have purpose.

P14

#### Perceptions of Increased Safety

Another subtheme related to how providers supported and justified the use of the system by discussing potential (but not yet implemented) enhancements to the safety of patients. Specifically, providers felt that the technology might allow them to intervene or react more efficiently to a presumptive risk with the potential to avoid injury. One person in the study expressed an interest in using the RTLS to reduce the use of restraints, while others mentioned their desire for proximity alarms that would alert providers when patients who are “having an altercation” are in close proximity to each other and allow them to intervene. The narratives of this theme include the following:

In terms of safety, like interactions with other...other patients...like you could, you know, set up some kind of parameters that alert the staff when my patient X gets within vicinity of patient Y.

P20

I think maybe even physically if you see that a patient has entered the washroom, an alert could go off into the care station. Um, there’s often times where I’ll find someone in there and they’re either beginning the process on their own and need help, or they are finishing the process and it’s become messy and there’s no way for us to know...But if an alert goes off in the care station, so and so has entered the bathroom, then whoever’s in the care station can respond to that before it gets messy.

P07

## Discussion

### Principal Findings

In this study, the introduction of an RTLS with restricted functionality of tracking the location of people and assets within a secure inpatient unit for persons living with dementia represents one of the most elementary implementations of a single technology to achieve a single task. The successful adoption of the technology was uncomplicated, and its uptake by HCPs was thus predictably swift. We aimed to investigate the factors influencing the adoption or nonadoption of the RTLS technology. We found that the successful adoption of the RTLS was due to the strong fit between the technology and the task, which was locating the patients more efficiently, and the strong individual and task relationship on the unit.

First, we summarize the results of the interactions between the 3 pillars of the FITT framework to explain the success of the implementation and identify the factors that influenced the adoption of the technology. Second, we address related issues that warrant consideration for similar and more complex implementation.

### Evaluating the FITT of the RTLS Implementation

The strong individual and task relationship on the unit was supported by an organization whose labor force was stressed due to the global pandemic and shortages and allied health and nursing HCPs who were aware of the challenges of ensuring that mobile individuals living with dementia were safe without constant one-to-one supervision. Individual providers who were responsible for locating patients in real time for events such as medication rounds, check-ins, patient visitors, and meals relied on installed closed-circuit television camera feeds. These cameras streamed to monitors mounted above the nursing station desk, providing real time but restricted line-of-sight hallway identification of patients and providers. However, the patient had to be in a hallway and remain in the same place long enough to be visualized. Depending on the patient, a care provider may need to search >1 bedroom to find a patient [22]. Providers’ anticipation of negative outcomes, both personally and to the patient from less than round-the-clock monitoring or to respond to risky behaviors such as interpersonal altercations or elopement, was a strong motivator not only to continue with what was described as an inefficient and time-consuming process but also to find and adopt a more efficient solution. As demonstrated in the narratives, the strong fit between the RTLS technology and the task was perceived as a clear benefit to staff and, as such, an influencing factor of technology uptake. When staff perceive that using the technology is the best fit for the completion of their tasks, they are more likely to adopt its use [23]. With this type of technology, Doshi-Velez et al [22] identified that being able to find patients more efficiently provides nursing staff with a strong motivation to use RTLS technology. Our HCPs provided examples of the perceived limitations of the current method of completing the patient locating. The time and energy required to find patients by “roaming” the halls were not generally perceived as a valuable use of time. Similar to research by Griffin et al [3], the HCPs appreciated the efficiencies that the technology afforded them (eg, the ability to “multitask” by reviewing the tablet app at the nursing station while completing another task, such as charting). HCPs mentioned that this was especially helpful during night shifts when HCP-to-patient ratios were lower (eg, fewer HCPs were responsible for more patients). Although HCPs were universally appreciative of reducing patient search time, they mentioned the indirect value of HCP “walkarounds” in the facility during patient searches. This informal “rounding” on the unit and attending to “distractions” over time and space was suggested by one participant as an opportunity for HCPs to intervene early or to prevent risky events or behaviors.

As the RTLS technology in this study passively *surveils*, it is neither predictive nor prescriptive; therefore, while it may help identify potential issues (eg, a patient alone in a bathroom or exiting the facility), with the functionality activated for this pilot, the system still required a provider to identify an issue (by visualizing patient location on the tablet), judge the potential risk, and determine if they needed to respond in person. The transactional (locating task) and anticipatory (monitoring task) use of the technology were both highly suited to the task of reducing risk for the individual, the institution, and the patient and provided peace of mind to visiting care partners who may perceive that the providers were more aware of the presence of their family or loved one.

Another influencing factor for adoption was the fit between the individual and the technology, partly due to the intrinsic capacity of the HCPs. We conducted foundational preinstallation training on the operation and use of the RTLS, and the HCPs were comfortable with workflow changes as a result of being a research-intensive facility (eg, by way of social influence and facilitating conditions), which has been found to be beneficial for the adoption of technology [24,25]. In addition, as found in the preinterview survey, the HCPs self-reported a positive attitude toward technology in general and the RTLS in particular. However, the fact that the technology was introduced to the staff as a pilot research project may have impacted its adoption. One HCP noted that if it had been presented as the de facto and permanent new method of locating patients, they would have been more invested in it.

Similar to Griffin et al [3], the HCP’s confidence in the RTLS system was based on trust. This included trust that the organization would not implement a system that did not secure patient location data, that the RTLS was reliable and presented accurate information, and that the organization’s trust in them as professionals should preclude the expansion of RTLS technology to monitoring staff for any reason. Similarly, the trust extended to the reliability that the system would be functional when needed. While the implementation of the RTLS presented few technological issues, beyond unplugged beacons, frozen screens, and drained bracelet batteries, that were unable to be managed by the on-site research coordinator (in addition to its fit with the unit workflow and operations), other issues emerged that are noteworthy.

### Perceptions of Safety

The decision to pilot or implement RTLS is frequently promoted as it has the potential to enhance unsupervised freedom of movement and improve the physical safety of persons living with dementia. Similar to the findings reported by Hall et al [26], the HCPs in our study identified that the primary rationale for using RTLS in this population was enhanced physical safety rather than freedom of movement. Providers alluded to technology-enhanced patient safety, yet it was difficult for them to articulate measurable outputs of related safety improvements compared to current interventions. It is important to distinguish between the “potential” of the technology’s capabilities and the actual functionality that helps realize improved safety. In reality, the functionality of the installed technology for our study was not “smart” nor was it predictive or responsive; unless a human was monitoring the app the moment a patient was at risk, there was no intervention or lessening of risk. The installed off-the-shelf technology in this study had both geofencing alerts and call button features but required human monitoring and sufficient staffing to respond and therefore were not enabled. Installations that enable these types of alerts (related to being outside a restricted area, experiencing a fall, or being in a location that suggests more risky behaviors) must be monitored to allow for a response in real time. This has resource implications related to technology (eg, mobile phone apps or tablets that allow remote monitoring rather than using a fixed desktop application) and human resources (eg, alerts must be responded to); thus, additional staff must be recruited to monitor, investigate, and respond to alerts anywhere on the floor at any time. Infrastructure and funding challenges and labor force shortages must be addressed to encourage more widespread exploration of the value and use of technologies such as RTLS in long-term care.

### Training and Adoption

HCPs in the study were oriented to the tablet app through training sessions, which focused on the features of the technology and its basic use, whether they voluntarily chose to use the location map app or not. The tablet was located beside the legacy surveillance camera feed, serving as a reminder of its availability. During the training sessions, those who were using the technology shared use cases and examples of when and how they found the system useful. At the start of the project, HCPs explained that their confidence in the system would be heightened if they could trust that it reliably showed the real location of patients and that the patients were unable to interfere with its operation. By the end of the study, most HCPs were using the tablet app based on their perception of its usefulness, its alignment with their comfort level with technology, and its ability to help them perform job responsibilities. As the pilot implementation progressed, “superusers” emerged [27,28], who were early adopters and who strongly advocated for its usefulness and value in the unit. Superusers often spontaneously helped others troubleshoot or navigate the RTLS and quickly identified alternative uses for the technology (eg, as asset tags). In one case, we downloaded a patient’s activity reports (a management feature of this particular RTLS), which helped to identify that they walked an extraordinary distance daily, allowing providers to integrate changes into the care plan (eg, encouraging rest times and increased caloric intake). Furthermore, some HCP users identified that the technology would be potentially more helpful if it were available on their phones or if there were more tablets accessible throughout the unit at different locations.

The setting for this study was a locked inpatient unit that already used sensors fitted to each patient to prevent their unaccompanied exit from the unit. Within the unit, the patients were free to move as they wished or were able to. As a short-stay unit, its focus was on stabilizing or addressing behaviors and facilitating patients’ return to the community. Unlike a long-term care home where residents’ mobility is often compromised, many of the patients in this unit were ambulatory; the organization’s interest in preserving patients’ dignity and aligning their values with those of patients by allowing their free movement in the unit had operational implications, such as unsupervised interactions between patients and increased provider time in seeking out patients for treatment and well-being checks. It is unsurprising that organizations are moving to replace what they believe to be non–value-added tasks performed by providers with technology-enhanced solutions such as RTLS [29]. Patients’ resistance to wearing the bracelet was reduced for some patients perceived the technology similar to an Apple Watch, which they described as “cool.” Providers suggested that to avoid dissent, further disguising the transponder, what Sannon and Forte [30] describe as “dignity in design” where aesthetics are considered along with utility, would provide more dignity to their patients when the organization and substitute decision makers’ values (to create a safe and risk-free environment) supersede patients’ right to privacy.

During the training of HCPs on the RTLS and in our research interviews, we noted a lack of in-depth discussion regarding the complexities and potential challenges associated with implementing monitoring technologies in a health care setting [26]. This approach, while simplifying the training and implementation process, may have allowed staff to focus on its potential to enhance patient safety but failed to engage the HCPs, care partners, and, where feasible, patients in a dialogue about the implications and challenges associated with its deployment, such as its impact on privacy. Hall et al [26] suggested that for technology implementations to be successful, they must involve substantive discussions to anticipate and address these challenges. Such inclusive conversations involving all stakeholders in the decision-making process are crucial for a more effective implementation of novel technological systems.

In the survey, all participants identified that using this technology to track persons with dementia was either somewhat (16/20, 80%) or completely (4/20, 20%) acceptable. During the interviews, the staff did not initiate any ethical concerns. Prompted questions related to the ethical use of ubiquitous monitoring technologies that collect sensitive biometric data, the creation of a surveillance culture, and the responsibility of organizations to respect the rights and dignity of susceptible individuals when using these technologies were not identified as a care priority by staff in the interviews. This finding has considerable risks and implications that have been explored in more detail elsewhere [2].

In the survey, the HCPs demonstrated a mixed level of acceptance of the RTLS being used to monitor HCP movement. This finding was echoed in the interviews, where HCPs expressed a hesitancy to use the technologies on themselves despite overall satisfaction with their work; this is consistent with previous studies [3,31], where workforce monitoring was viewed as a lack of management-worker trust, a manifestation of the blame culture, and a foot in the door where monitoring for quality of care (eg, time spent with patients or handwashing) would be the first step to its use for individual performance tracking (eg, length of breaks and productivity). The shift of health care administration, toward the adoption of more scientific management where standardization, removal of inefficiencies, and process improvement, supports the notion that RTLS might be used in this fashion [32]. Overt messaging from the management that the monitoring in this study was limited to patients supported its successful adoption and implementation.

### HCP Workload and Technology Support

Technology has increased the amount and complexity of information that employees are expected to process and has enabled HCPs to access unlimited amounts of information to do their jobs [33]. In the health care environment in which provider resources are scarce, workloads are high. Some HCPs on the floor did not use the RTLS (and did not volunteer to participate in our study); those who participated cited its simplicity, reliability, and ability to reduce their perceived workload as the reason for their continued use of the technology, rather than the rationale of safety as the rationale for adoption. Workload presented a challenge in relation to troubleshooting technological issues with the RTLS. Although providers were comfortable with the RTLS, none moved into a role where they were able to troubleshoot simple technological issues. Most cited the lack of time, the availability of a low-cost alternative (walking around), and the presence of a research team for technological support; however, this support was not available 24/7.

### Limitations

The generalization of findings from data collected at a single location during one technology implementation has limitations. However, our focus on exploring the perspectives of HCPs working in an environment that provides specialized dementia care was both a strength and a limitation. The perspectives of the organizational decision makers on the rationale for adopting and implementing the RTLS technology in the unit were outside the scope of the study. These findings will be important to explore in future research on the adoption of RTLS in dementia care settings.

Providers’ self-reported comfort with the installed RTLS was evident in the preinterview questionnaire responses and after providers were trained and had used the technology. Furthermore, the unit under study is a teaching and research-intensive hospital that frequently involves providers in technology-enabled research studies. The uniqueness of the unit may have impacted the self-reported providers’ job satisfaction where, particularly during the pandemic, restricted access to the unit and the limited number of patients and providers offered some immunity from the ongoing pandemic pressures related to staffing shortages, frequent outbreaks, and increased workloads, which were experienced in other congregate settings for people living with dementia [34].

The staff members on the unit were acquainted with the principal investigator and the research coordinator of this study, who conveyed the study’s purpose to the participants, responded to staff inquiries, and obtained participant consent. To ensure impartiality, the study team used deductive analysis of the data and applied the FITT framework as a standardized approach to analyze the data. This framework is a well-established and widely used approach in the literature, which helped to reduce the potential for researcher bias in data interpretation. Furthermore, the fact that the unit uses other technology, such as the elopement prevention system, suggests that staff members are accustomed to using technology in their daily work routine. This familiarity with technology may have helped to reduce any bias toward or against the RTLS technology used in this study.

Another limitation relates to the small scale of this study. Although the number of participants was sufficient to reach saturation with respect to our research questions, it is insufficient to generalize beyond the specific context of the featured location-tracking technology, its functionality, and the hospital-based dementia care setting in which it was implemented.

### Conclusions

Similar to many novel technologies in the nascent stages of their adoption, evidence to support the utility and effectiveness of RTLS in improving the safety and quality of care in health care organizations and patients’ experience is limited. While HCPs were able to reduce their self-reported patient search time dramatically, sometimes by half, any improvements in care were implied or perceived. While no participants self-reported evidence of reduced patient risk, reduced adverse events, or improved outcomes, they described greater perceived peace of mind for the staff responsible for oversight. As stewards of resource-constrained pragmatic organizations, decision makers in the health care sector will weigh the risks of RTLS adoption related to personal privacy, overreliance on untested technology, and cost against the benefits of ubiquitous monitoring of human and equipment assets, performance management, and automation of location tasks to improve staff efficiency. The decision to adopt novel technologies necessitates examination policies, workflows, and resource commitments beyond the initial purchase costs of the hardware, software, and training to identify technologies and adoption processes that best fit the organizational context and the tasks it must perform.

## Appendix

Multimedia Appendix 1 Interview guide.

## Abbreviations

FITT: Fit Between Individuals, Tasks, and Technology
HCP: health care provider
REDCap: Research Electronic Data Capture
RTLS: real-time location system
UHN: University Health Network
