# Supplementary material for: Factors That Influence Successful Adoption of Real-Time Location Systems for Use in a Dementia Care Setting: Mixed Methods Study
Source: JMIR Aging. 2024 Apr 8;7:e45978. doi: 10.2196/45978 (PMC11036182; doi:10.2196/45978)
Supplement: Multimedia Appendix 1 [file aging_v7i1e45978_app1.docx]

**STICS Pilot Sub-study: Interview Guide**

Thank you for participating in this interview. I would like to remind you that you are free to withdraw from the study at any time, and similarly if you do not wish to respond to a question just indicate by saying “pass”.

1. Have you ever used RTLS technology such as Tenera before?
   - 1. If yes,
        - What was the technology and in what context was it being used?
        - Did that experience influence whether or not you used/are using Tenera?
2. **Goals and expectations** regarding RTLS technologies such as Tenera:
   1. What do you see as the main purpose of this type of technology?
      1. What do you like about this?
      2. What don’t you like about it?
   2. Thinking back to when you were making the decision to use or not use the Tenera technology:
      1. What were the main factors that influenced your decision to use the system or not?
      2. What were your thoughts when you first saw the implementation of the system? (did you know what it was for? What did you expect this would mean for you?)
      3. Thinking about your normal workflows - how did you think you might or might not have had to change your workflow to integrate the use of a new technology?
      4. Can you tell me how you considered your workload might or might not change with the system?
   3. What impact did others using or not using the technology have on your decision?

Prompts:

- Think about this type of technology and your beliefs that it can perform certain functions; have you had experiences with other similar technologies that influenced your decision to use the system?
- Consider your level of comfort with technology generally; do you think this influenced your decision to use the system? In what way?
  1. Was Tenera useful for other staff on the unit?
     1. Can you tell me how other staff are using the system? Has this influenced the way that you use the system?

**User Experiences**

1. *<<Only for those who used the system>*
   - 1. Can you tell me about at time that you weren’t able to find a patient before using Tenera?
        - How long did it normally take to locate patients (from beginning to end?)
        - On average in a week, how long do you think you would spend looking for someone?
        - Was the way you located patients normally changed when the Tenera system was installed?
        - How did it change?
2. How often did you use Tenera?
   - - - Could you estimate how long it takes you to use the Tenera system to find a patient instead of looking at the video cameras?
       - Has the use of Tenera impacted your workflow? How so?
3. How useful do you think this tool will be/was for your work?
   - - Locating patients in real-time?

- Was it more useful with some patients than others?
- What is needed to enhance its usefulness?
- Are you using it for any other purpose?

1. Can you tell me how reliable it was in locating patients?
2. What benefits did you experience from using Tenera?
3. What challenges did you experience using Tenera?
4. Have you found Tenera effective/useful for clinical care?
   - - - *Why or why not?*

**Relationships:**

1. What do you think is the impact of (using) Tenera on patients?
   1. And the impact of the system for families/care partners? Care providers?
   2. What is the impact of Tenera on your relationships with patients, between staff, etc?
      - - How might positive impacts be optimized, negative impacts mitigated?
        - Did using Tenera impact your relationships with patients/ families/care partners/co-workers/others?

**Other considerations**

1. We are going to talk about potential areas of concern about using this system. Overall, do you have any concerns/worries regarding the use of this type of technology? Tenera in particular?

*Prompt:*

- - *Can you tell me about any concerns or worries you would have about using the technology for continuous monitoring of patients’ cognitive or physical decline?*
  - *How would you feel about using Tenera for monitoring of staff movement and/or activities?*
  - *Do you have any privacy concerns for your patients or yourself?*
  - *How do you feel about the security of the data collected?*
  - *How do you feel about potential Ethical issues related to the patients’ inability to consent?*
  - *Can you tell me about how you feel about the lack of evidence to support the value of continuous monitoring?*
  - *Have you found any instances of patient discomfort/resistance? How would you deal with this situation?*

1. Did the Tenera system meet your expectations?
   - Can you share any examples of challenges or negative events that you may have experienced with the system? (e.g. resistance/discontinuation and other problems it created in use, technology access, workflow)
   - Can you share any examples of positive events and suggestions for improvement (e.g. incentives, changes needed)
2. How would you change the technology to make it work better/be more useful for you?
   - - - 1. Based on your experience using Tenera, would you recommend this technology to a respected colleague, family or friends?

*[Please explain]*

1. How might Tenera be used in the future to monitor the physical, cognitive and mental wellbeing of residents?

*[Prompt: add a call button for assistance, have it on mobile technology so you don’t have to go to nursing desk*

1. Do you have any concerns regarding the use of RTLS technology or Tenera in dementia care settings that we haven’t already discussed today?

**Implementation**

1. What do you feel were/are the key challenges to the implementation of Tenera on your unit? On other units?
2. Based on your experiences, what do you feel might be key **challenges** to the implementation of an RTLS like Tenera in residential dementia care settings such as nursing homes? (things that could make implementation easier)

- *Prompt: How might these be minimized/overcome? Do you feel that implementation in these settings will be different than on the unit? If yes, why?*

1. What do you feel might be the key **facilitators** to the implementation of an RTLS like Tenera in residential dementia care settings such as nursing homes? (things that could make implementation harder?)

Thank you for your time today. We will follow up with your gift card information via email; if you are interested in providing incremental insights or follow up comments during this study after your interview ends, please contact me at my email.
